# High absolute lymphocyte counts are associated with longer overall survival in patients with metastatic breast cancer treated with eribulin—but not with treatment of physician’s choice—in the EMBRACE study

**DOI:** 10.1007/s12282-020-01067-2

**Published:** 2020-03-05

**Authors:** Yasuo Miyoshi, Yuta Yoshimura, Kenichi Saito, Kenzo Muramoto, Michiko Sugawara, Karenza Alexis, Kenichi Nomoto, Seigo Nakamura, Toshiaki Saeki, Junichiro Watanabe, Jose Manuel Perez-Garcia, Javier Cortes

**Affiliations:** 1 Department of Surgery, Division of Breast and Endocrine Surgery, Hyogo College of Medicine, Mukogawa-cho 1-1, Nishinomiya, Hyogo 663-8501 Japan; 2 Eisai Co., Ltd., Koishikawa, Bunkyo-ku, Tokyo Japan; 3 Eisai Inc., Woodcliff Lake, NJ USA; 4 Division of Breast Surgical Oncology, Department of Surgery, Showa University, Hatanodai, Shinagawa-ku, Tokyo Japan; 5 Department of Breast Oncology, International Medical Center, Saitama Medical University, Yamane, Hidaka, Saitama, Japan; 6 Division of Breast Oncology, Shizuoka Cancer Center, Shimonagakubo, Nagaizumi-cho, Sunto-gun, Shizuoka, Japan; 7 IOB Institute of Oncology, Quironsalud Group, Madrid, Barcelona, Spain; 8 Medica Scientia Innovation Research (MedSIR), Barcelona, Spain; 9 Breast Cancer Group, Vall D’Hebron Institute of Oncology (VHIO), Barcelona, Spain

**Keywords:** Eribulin, Absolute lymphocyte count, Overall survival, Metastatic breast cancer, Treatment of physician’s choice

## Abstract

**Background:**

Eribulin, a nontaxane synthetic inhibitor of microtubule dynamics, is widely used to manage locally advanced or metastatic breast cancer (MBC). Eribulin has demonstrated immunomodulatory activity on the tumour microenvironment. Baseline neutrophil-to-lymphocyte ratio (NLR), a marker of immune status, may predict progression-free survival in eribulin treatment. This post hoc analysis assessed predictors for overall survival (OS).

**Methods:**

The phase 3 open-label study (EMBRACE) of eribulin versus treatment of physician’s choice (TPC) in patients with MBC provided source data. Baseline absolute lymphocyte counts (ALCs) and NLR were evaluable in 751 and 713 patients, respectively.

**Results:**

Eribulin prolonged OS versus TPC in patients with baseline ALC ≥ 1500/µl (hazard ratio [HR] 0.586; 95% confidence interval [CI] 0.437–0.784; *P* < 0.001). There was no significant difference by treatment for ALC < 1500/µl (HR 1.002; 95% CI 0.800–1.253; *P* = 0.989). Univariate and multivariate analyses were performed and identified baseline ALC as a potential predictor of OS in eribulin-treated patients. Interaction analysis of OS supported 1500/µl as a potentially differential cutoff value. NLR at a cutoff value of 3 was associated with prolonged OS (eribulin group). However, similar results were also observed in the TPC group, without apparent interaction effect, suggesting that NLR may be a general prognostic marker rather than a specific predictor of OS for eribulin.

**Discussion:**

This hypothesis-generating study speculates that baseline ALC may be an independent predictor for longer OS in eribulin-treated MBC patients and could be clinically impactful because it can be evaluated without the need for additional invasive procedures.

**Trial Registration:**

www.ClinicalTrials.gov code: NCT00388726

**Electronic supplementary material:**

The online version of this article (10.1007/s12282-020-01067-2) contains supplementary material, which is available to authorized users.

## Introduction

Eribulin, a synthetic inhibitor of microtubule dynamics, is widely used for locally advanced or metastatic breast cancer (MBC) after one or two previous lines of chemotherapy in Europe and the United States, respectively [1, 2]. In Japan, eribulin has been approved for inoperable or recurrent breast cancer, following treatment with an anthracycline and a taxane [3]. Eribulin monotherapy versus treatment of physician’s choice (TPC) in patients with MBC (EMBRACE) was a phase 3, open-label, randomised study of 762 patients (eribulin, *n* = 508; TPC, *n* = 254) [4]. Overall survival (OS) was significantly longer in the eribulin group versus the TPC group (median OS: 13.1 vs 10.6 months, respectively; hazard ratio [HR] 0.81; 95% confidence interval [CI], 0.66–0.99; *P* = 0.041). However, there was no statistically significant difference in progression-free survival (PFS). Although the impact of eribulin on OS distinguishes it from other neoplastic agents (including taxanes) in pretreated MBC, the exact mechanism behind this difference in efficacy outcomes is not well understood.

In addition to its antimitotic effect, eribulin facilitates antineoplastic activity in MBC by reversing epithelial-to-mesenchymal transition (EMT) and inducing vascular remodelling [5, 6]. Kashiwagi et al. [7] reported that the number of tumour-infiltrating lymphocytes (TILs) in the tumour microenvironment may be a predictor of eribulin’s therapeutic effect in patients with triple-negative MBC; patients with higher numbers of TILs had significantly longer PFS than patients with lower numbers of TILs. In a single-institute retrospective study, Miyagawa et al. [8] demonstrated that neutrophil-to-lymphocyte ratio (NLR), a marker of systemic immunity, was significantly associated with PFS in eribulin-treated patients but not in those treated with nab-paclitaxel for MBC. Median PFS of patients with a baseline NLR < 3 was significantly longer than patients with a baseline NLR ≥ 3 (242 days vs 98 days; HR 0.37; 95% CI 0.18–0.71) [8].

NLR and TILs are markers of immunological status associated with predicting outcomes in patients with cancer [9–13]. Therefore, we speculate that eribulin’s ability to mediate immunological regulation in patients with MBC may clarify why there is a greater impact on OS compared with PFS. This post hoc analysis used data from EMBRACE to evaluate absolute lymphocyte count (ALC) and NLR as predictors of OS with eribulin.

## Patients and methods

### Clinical study design

The study design and primary efficacy and safety results of EMBRACE have been reported previously [4]. Briefly, patients with ≥ 2 previous chemotherapy regimens (including an anthracycline and taxane, for locally recurrent breast cancer or MBC) were randomised 2:1 to receive eribulin or TPC. Patients with baseline ALC and NLR assessments (i.e., the last nonmissing result prior to the first administration of study drug) were included in the analysis for association between baseline ALC/NLR and OS/PFS outcomes. Over 90% of baseline blood samples were collected within 3 days prior to initial administration of eribulin or TPC. If these blood samples were unavailable, blood samples obtained during screening were utilised.

Approval was obtained from independent ethics committees and regulatory authorities in participating countries, and all patients provided written informed consent. EMBRACE was conducted in accordance with the World Medical Association Declaration of Helsinki (WMA General Assembly, Tokyo, 2004) guidelines of the Committee for Proprietary Medicinal Products/International Conference for Harmonisation/Good Clinical Practice (CPMP/ICH/135/95) and local ethical/legal requirements.

### Treatments

Eribulin was administered intravenously at a dose of 1.4 mg/m^2^ during a 2–5-min infusion on days 1 and 8 of a 21-day cycle. TPC was administered according to local practice; both eribulin and TPC continued until disease progression, unacceptable toxic effects, patient or physician request to discontinue, or serious protocol noncompliance [4].

### Post hoc analysis of patient outcomes

OS was measured from the date of randomisation to the date of death from any cause. OS was censored at the last date that patients were known to be alive. PFS was defined as the time from the date of randomisation to the date of radiological disease progression based on independent review or death from any cause, whichever occurred first. PFS was censored at the date of last radiological assessment.

### Statistical analyses

The Kaplan–Meier method was used to estimate OS/PFS distribution. The cutoff value for baseline ALC and NLR was set at 1500/µl [14] and 3 [8], respectively. HRs of the high-ALC or low-NLR group versus the low-ALC or high-NLR group were estimated from the stratified Cox proportional hazard model with ALC or NLR group as an independent variable and stratified by the randomisation stratification factors: HER2/neu status, prior capecitabine treatment, and geographical region. Subgroup analyses were conducted in a similar manner. Differences between groups were evaluated using the stratified log-rank test with randomisation stratification factors. Interactions between treatment and baseline ALC/NLR were explored using a stratified Cox proportional hazard model with treatment, ALC/NLR category, and their interaction, as covariates. For baseline ALC, the interactions were also explored broadly with different cutoff values. Univariate/multivariate analyses for baseline factors were performed using the Cox proportional hazard model to identify predictors for OS. Backward selection was used with a significance level of 10% for retaining the factors in the multivariate model. Multiplicity adjustments were not used for any analyses. Analyses were performed using SAS version 9.3 (SAS Institute Inc., Cary, NC, USA).

## Results

### Patient characteristics

762 Patients were enrolled in the intent-to-treat population and were randomised to receive eribulin (*n* = 508) or TPC (*n* = 254). There were evaluable baseline ALC results for 500 (eribulin) and 251 (TPC) patients (751 total; Online resource Fig. 1). Baseline characteristics were similar between groups with ≥ 1500/µl (high ALC) and < 1500/µl (low ALC) in both treatment groups with the exception of the number of patients with > 2 organs involved (high ALC; 19–24% vs low ALC; 30–39%) and the number of patients from North America/Western Europe/Australia (high ALC; 53–55% vs low ALC; 69–70%) (Table 1). There were evaluable baseline NLR results for 475 (eribulin) and 238 (TPC) patients (713 total). Baseline characteristics were similar between ≥ 3 (high NLR) and < 3 (low NLR) patients in both the eribulin and TPC groups except for the number of patients with > 2 organs involved (high NLR; 32–41% vs low NLR; 20–26%), > 3 prior chemotherapy regimens (high NLR; 60–62% vs low NLR; 47–50%) and the number of patients from North America/Western Europe/Australia (high NLR; 74–75% vs low NLR; 57–60%) (Online Resource Table 1). Patients were heavily pretreated (median, 4 previous chemotherapy regimens). Median (interquartile range) baseline ALCs for eribulin, TPC, and overall groups were 1308 (1000, 1814), 1307 (991, 1697), and 1307 (1000, 1776), respectively. Median (interquartile range) baseline NLRs for eribulin, TPC, and overall groups were 3.05 (2.13, 4.44), 3.06 (2.14, 4.19), and 3.05 (2.14, 4.41), respectively.

**Table 1:** Demographic and baseline characteristics by ALC group

Characteristic, *n* (%)	Eribulin (*n* = 500)		TPC^a^ (*n* = 251)	
	ALC ≥ 1500/μl (*n* = 199)	ALC < 1500/μl (*n* = 301)	ALC ≥ 1500/μl (*n* = 92)	ALC < 1500/μl (*n* = 159)
Geographical region				
North America/Western Europe/Australia	106 (53)	212 (70)	51 (55)	110 (69)
Eastern Europe	66 (33)	62 (21)	29 (32)	34 (21)
Latin America/South Africa	27 (14)	27 (9)	12 (13)	15 (9)
HER2 status				
Positive	34 (17)	48 (16)	16 (17)	23 (14)
Negative	146 (73)	221 (73)	69 (75)	121 (76)
Unknown	19 (10)	32 (11)	7 (8)	15 (9)
Prior capecitabine treatment				
Yes	136 (68)	228 (76)	65 (71)	122 (77)
No	63 (32)	73 (24)	27 (29)	37 (23)
ECOG performance status				
0	87 (44)	127 (42)	40 (43)	63 (40)
≥ 1	110 (55)	168 (56)	51 (55)	94 (59)
Age group				
< 65 years	166 (83)	242 (80)	76 (83)	120 (75)
≥ 65 years	33 (17)	59 (20)	16 (17)	39 (25)
ER status				
Positive	136 (68)	195 (65)	55 (60)	114 (72)
Negative	47 (24)	95 (32)	33 (36)	39 (25)
Unknown	16 (8)	11 (4)	4 (4)	6 (4)
PgR status				
Positive	103 (52)	148 (49)	43 (47)	78 (49)
Negative	68 (34)	126 (42)	37 (40)	65 (41)
Unknown	28 (14)	27 (9)	12 (13)	16 (10)
HR status				
Positive	141 (71)	203 (67)	61 (66)	116 (73)
Negative	38 (19)	85 (28)	26 (28)	37 (23)
Unknown	20 (10)	13 (4)	5 (5)	6 (4)
Triple negative				
Triple negative	27 (14)	65 (22)	21 (23)	30 (19)
Non-triple negative	172 (86)	236 (78)	71 (77)	129 (81)
Site of disease				
Visceral disease	160 (80)	247 (82)	74 (80)	135 (85)
Nonvisceral disease	36 (18)	52 (17)	17 (18)	22 (14)
Number of organs involved				
≤ 2	159 (80)	208 (69)	69 (75)	95 (60)
> 2	37 (19)	91 (30)	22 (24)	62 (39)
Number of prior chemotherapy regimens				
≤ 3	111 (56)	127 (42)	44 (48)	69 (43)
> 3	88 (44)	172 (57)	48 (52)	89 (56)
Number of prior chemotherapy regimens for locally advanced or metastatic disease				
≤ 3	165 (83)	221 (73)	66 (72)	112 (70)
> 3	34 (17)	80 (27)	26 (28)	46 (29)
Refractory to taxanes^b^				
Yes	154 (77)	249 (83)	76 (83)	127 (80)
No	45 (23)	52 (17)	16 (17)	32 (20)

*ALC* absolute lymphocyte count, *ECOG* Eastern Cooperative Oncology Group, *ER* oestrogen receptor, *HER2* human epidermal growth factor receptor-2, *HR* hormone receptor, *PgR* progesterone receptor, *TPC* treatment of physician’s choice

^a^TPC was defined as any single-agent chemotherapy, or hormonal or biological therapy, approved for the treatment of cancer

^b^Disease progression on or within 6 months of taxane treatment

### OS and PFS outcomes

**Fig. 1:**
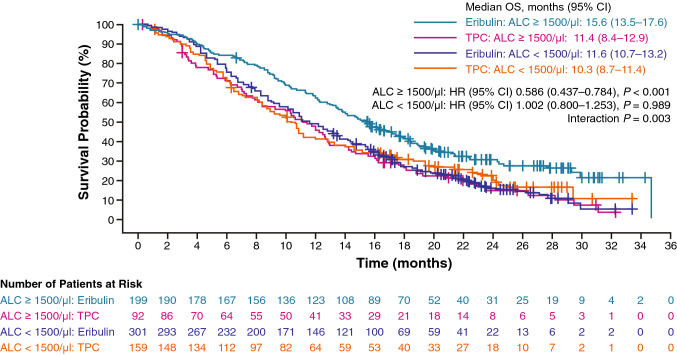
Interaction between treatment and baseline ALC (< 1500/µL vs ≥ 1500/µL) on OS. In patients with high ALC, the median OS was 15.6 and 11.4 months in the eribulin and TPC arms, respectively (HR 0.586; 95% CI 0.437–0.784; *P* < 0.001). In patients with low ALC, the median OS was 11.6 and 10.3 months in the eribulin and TPC arms, respectively (HR 1.002; 95% CI 0.800–1.253; *P* = 0.989). *ALC* absolute lymphocyte count, *CI* confidence interval, *HR* hormone receptor, *OS* overall survival

Using the cutoff value of ALC (“high” ≥ 1500/μl and “low” < 1500/μl), OS was compared in patients treated with eribulin (high ALC, *n* = 199 [40%]; low ALC, *n* = 301 [60%]) versus TPC (high ALC, *n* = 92 [37%]; low ALC, *n* = 159 [63%]). OS was prolonged in the eribulin versus the TPC group in patients with high ALC (median 15.6 months vs 11.4 months; HR 0.586; 95% CI 0.437–0.784; *P* < 0.001); in patients with low ALC, the difference was not significant (median, 11.6 months vs 10.3 months; HR 1.002; 95% CI 0.800–1.253; *P* = 0.989) (Fig. 1). Moreover, statistical signals of an interaction effect between treatment and baseline ALC were found; OS was longer in the high-ALC group versus the low-ALC group (HR 0.631, 95% CI 0.505–0.789) with eribulin but not TPC. Although this association was found in PFS within each treatment, there were no significant differences in PFS between the eribulin and TPC arms in either the high- or low-ALC groups (Online Resource Fig. 2).

**Fig. 2:**
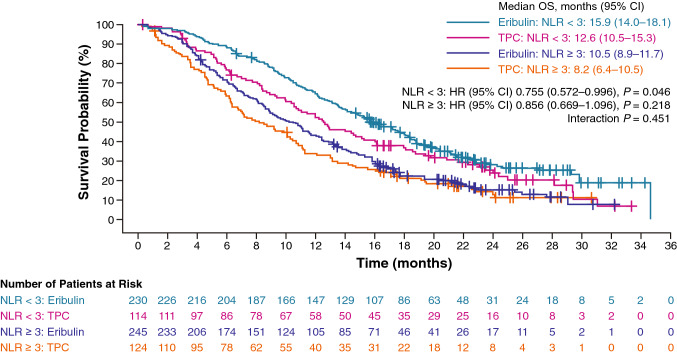
Interaction between treatment and baseline NLR (< 3 vs ≥ 3) on OS. In patients with high NLR, the median OS was 10.5 and 8.2 months in the eribulin and TPC arms, respectively (HR 0.856; 95% CI 0.669–1.096; *P* = 0.218). In patients with low NLR, the median OS was 15.9 and 12.6 months in the eribulin and TPC arms, respectively (HR 0.755; 95% CI 0.572–0.996; *P* = 0.046). *CI* confidence interval, *OS* overall survival

OS was also compared using an NLR cutoff value of 3 (“high” ≥ 3 and “low” < 3) in patients treated with eribulin (high NLR, *n* = 245 [52%]; low NLR, *n* = 230 [48%]) vs TPC (high NLR, *n* = 124 [52%]; low NLR, *n* = 114 [48%]). OS was prolonged with eribulin versus the TPC group in patients with low NLR (median, 15.9 months vs 12.6 months; HR 0.755; 95% CI: 0.572–0.996; *P* = 0.046); in patients with high NLR, the difference was not significant (median, 10.5 months vs 8.2 months; HR 0.856; 95% CI 0.669–1.096; *P* = 0.218). There were no statistical signals of an apparent interaction effect between treatment and baseline NLR (Fig. 2). OS was longer in the low-NLR group versus the high-NLR group within each treatment; a similar association was also found for PFS (Online Resource Fig. 3).

### Univariate/multivariate analyses of baseline factors for OS

Univariate/multivariate analyses were conducted to explore baseline predictors of OS and confirm baseline ALC as a predictor of OS, adjusting for other potential confounding factors (Table 2). Six parameters were identified as predictors of OS in the eribulin group: (1) prior capecitabine use, (2) ECOG performance status, (3) HR status, (4) number of organs involved, (5) refractory to taxanes, and (6) baseline ALC. In the TPC group, four of these factors (with the exception of prior capecitabine use and baseline ALC) affected OS.

**Table 2:** Univariate/ multivariate analyses of the baseline factors for OS in EMBRACE

Parameter	Eribulin				TPC			
	Univariate		Multivariate		Univariate		Multivariate	
	Hazard Ratio (95% CI)	*P*-value	Hazard Ratio (95% CI)	*P*-value	Hazard Ratio (95%CI)	*P*-value	Hazard Ratio (95% CI)	*P*-value
North America/Western Europe/Australia vs Latin America/South Africa	0.783 (0.568–1.079)	0.1349	–	–	0.845 (0.531–1.343)	0.4757	–	–
Eastern Europe (vs Latin America/South Africa)	0.736 (0.513–1.055)	0.0951	–	–	0.703 (0.420–1.175)	0.1786	–	–
HER2 status (positive vs negative)	1.106 (0.845–1.446)	0.4630	–	–	1.387 (0.964–1.995)	0.0783	–	–
Prior capecitabine (yes vs no)	1.262 (1.000–1.593)	0.0497	1.292 (1.006–1.660)	0.0447	1.316 (0.954–1.816)	0.0942	–	–
ECOG performance status (0 vs ≥ 1)	0.593 (0.482–0.729)	< 0.0001	0.598 (0.481–0.745)	< 0.0001	0.526 (0.394–0.704)	< 0.0001	0.563 (0.418–0.758)	0.0002
Age group (< 65 years vs ≥ 65 years)	0.904 (0.705–1.160)	0.4278	–	–	1.307 (0.924–1.848)	0.1301	–	–
ER status (positive vs negative)	0.726 (0.582–0.906)	0.0047	–	–	0.815 (0.599–1.110)	0.1946	–	–
PgR status (positive vs negative)	0.795 (0.644–0.982)	0.0331	–	–	1.079 (0.804–1.449)	0.6114	–	–
HR status (positive vs negative)	0.664 (0.528–0.834)	0.0004	0.649 (0.512–0.822)	0.0003	0.770 (0.557–1.066)	0.1151	–	–
Triple negative (vs non-triple negative)	1.475 (1.148–1.895)	0.0023	–	–	1.305 (0.924–1.843)	0.1309	–	–
Site of disease (visceral vs nonvisceral)	1.265 (0.960–1.667)	0.0952	–	–	1.479 (0.992–2.207)	0.0550	–	–
Number of organs involved (≤ 2 vs > 2)	0.615 (0.493–0.766)	< 0.0001	0.559 (0.442–0.707)	< 0.0001	0.615 (0.462–0.818)	0.0009	0.689 (0.514–0.925)	0.0132
Number of prior chemotherapy regimens (≤ 3 vs > 3)	0.870 (0.711–1.063)	0.1734	–	–	0.865 (0.654–1.144)	0.3102	–	–
Number of prior chemotherapy regimens for locally advanced or metastatic disease (≤ 3 vs > 3)	0.752 (0.599–0.944)	0.0142	–	–	0.817 (0.606–1.103)	0.1868	–	–
Refractory to taxanes^a^ (yes vs no)	1.682 (1.283–2.206)	0.0002	1.694 (1.267–2.264)	0.0004	0.912 (0.649–1.283)	0.5980	–	–
Baseline ALC (≥ 1500/μL vs < 1500/μL)	0.670 (0.542–0.827)	0.0002	0.761 (0.607–0.955)	0.0183	1.098 (0.826–1.459)	0.5208	–	–

*ALC* absolute lymphocyte count, *CI* confidence interval, *ECOG* Eastern Cooperative Oncology Group, *ER* oestrogen receptor, *HER2* human epidermal growth factor receptor-2, *HR* hormone receptor, *PgR* progesterone receptor

^a^Disease progression on or within 6 months of taxane treatment

### Subgroup analyses for baseline ALC effects on OS

**Fig. 3:**
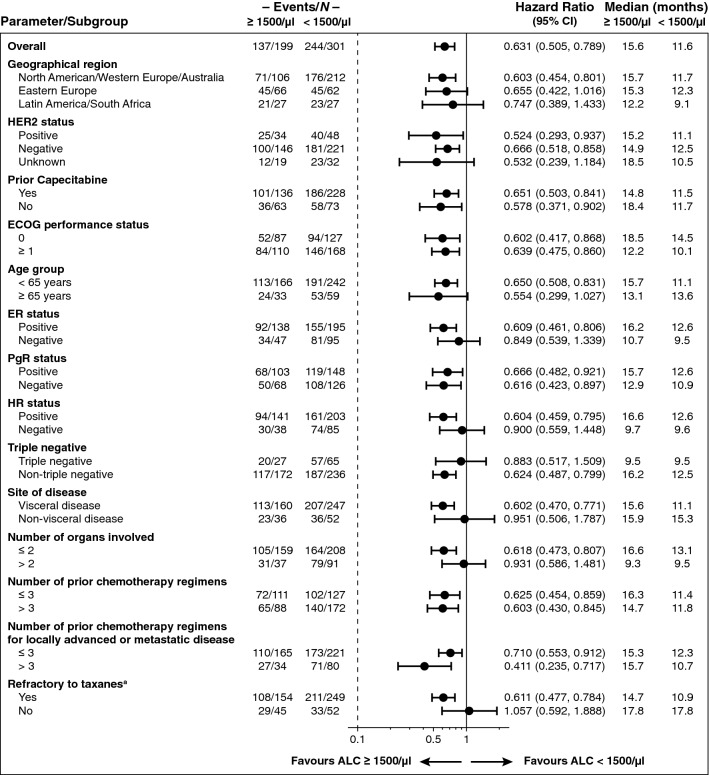
The forest plot for baseline ALC (< 1500/µl vs ≥ 1500/µl) effects on OS for the eribulin arm indicates that high ALC consistently favoured OS in all parameters analysed, except for patients who were not refractory to taxanes. *ALC* absolute lymphocyte count, *CI* confidence interval, *ECOG* Eastern Cooperative Oncology Group, *ER* oestrogen receptor, *HER2* human epidermal growth factor receptor-2, *HR* hormone receptor, *OS* overall survival, *PgR* progesterone receptor. ^a^Disease progression on or within 6 months of taxane treatment

Subgroup analysis on OS for baseline ALC (≥ 1500/μl vs < 1500/μl) in patients treated with eribulin indicated that high ALC consistently favoured OS in all parameters analysed, with the exception of patients not refractory to taxanes (Fig. 3). In patients treated with TPC, the subgroup analysis did not demonstrate a consistent correlation between high ALC and OS (Online Resource Fig. 4).

### Cutoff value of ALC

An interaction analysis of OS was performed between treatment and baseline ALC in EMBRACE to evaluate baseline ALC as a predictive factor of eribulin’s effect and to confirm the cutoff values of ALC (Online Resource Table 2). Numerically longer median OS was observed in the eribulin group compared with the TPC group regardless of baseline ALC. However, HRs of eribulin versus TPC in the high-ALC groups were consistently lower than those in the low-ALC groups. Moreover, the benefits of eribulin in high-ALC groups were greater than in the low-ALC groups across the cutoff values of 1400–1700/μl (interaction *P* < 0.05). Notable differential effects were observed around cutoff values of 1500/μl (interaction *P* = 0.003). The HR of eribulin versus TPC was 0.586 (95% CI 0.437–0.784) in patients with baseline ALC ≥ 1500/μl.

## Discussion

In this post hoc analysis conducted with data from EMBRACE, a high ALC (≥ 1500/µl) was found to be a significant and independent predictor for longer OS in patients treated with eribulin, but not in those treated with TPC.

Previously, Miyagawa et al. [8] found that NLR was significantly associated with PFS in eribulin-treated patients. Recently, Araki et al. [14] reported that baseline ALC at a cutoff value of 1500/µl is a predictor for PFS in HER2-positive advanced breast cancer treated with pertuzumab and trastuzumab, irrespective of combination chemotherapy regimens including eribulin. Collectively, these results suggest that the status of the tumour microenvironment affects the efficacy of eribulin. Therefore, this analysis focused on baseline ALC and NLR as peripheral immunological biomarkers associated with the efficacy of eribulin. In EMBRACE [4], at a cutoff value of 3, NLR was associated with prolonged PFS and OS in the eribulin group. However, similar results were also observed in the TPC group, without apparent interaction effect. This suggests that NLR may be a general prognostic marker rather than a specific predictor of OS for eribulin, although NLR cannot be excluded as a potential predictor for other agents. Therefore, it seems that ALC might be a superior predictive marker of improved OS with eribulin than NLR. Based on this analysis, ALC appears to be a specific predictor of OS for eribulin, but not a general prognostic factor. To the best of our knowledge, this is the first report demonstrating ALC as a predictive marker for OS benefit in MBC. Considering these outcomes, baseline ALC may be used as a specific biomarker to predict the survival benefit conferred by eribulin treatment in patients with MBC.

Eribulin induced the remodelling of the tumour vasculature in a preclinical xenograft model and in patients with breast cancer in several studies [6, 15–17]. Additionally, eribulin induced reoxygenation by vascular remodelling in patients with advanced breast cancer and decreased transforming growth factor-beta (TGF-β), which is typically associated with hypoxic conditions [15]. In a retrospective analysis of eribulin responders (patients with MBC), programmed cell death-1 (PD-1), programmed cell death ligand-1 (PD-L1), and forkhead box P3 levels reportedly decreased, while infiltrated CD8+ T-cell levels increased, but these findings were not observed in the nonresponder group [18]. As PD-1/PD-L1 pathways and TGF-β have potent immunosuppressive effects [19], these results suggested that eribulin may have had an immunomodulatory effect mediated through vascular remodelling, especially in the responder group.

Other research has demonstrated that baseline ALC was associated with longer OS in melanoma patients treated with ipilimumab, an anti-CTLA-4 antibody [20]. Ku et al. [21] reported a trend towards improved OS in a high-ALC group in patients with melanoma treated with ipilimumab (median OS, 13.3 vs 5.1 months; *P* = 0.06). Ku et al. concluded that baseline ALC has the potential to be an immunological predictive index for OS when treated with immune checkpoint inhibitors (ICIs).

Considering that ALC may be predictive of the efficacy of ICIs, ALC may potentially reflect the immune microenvironment within the tumour. Reportedly, hypoxia induces an increase in the expression of PD-L1 via the hypoxia-inducible factor-1 (HIF-1) transcription factor in myeloid-derived suppressor cells [22]. HIF-1 also regulated TGF-β, which has potent immunosuppressive effects and promotes the growth of breast cancer. TGF-β also plays a role in EMT [23]. As eribulin may potentially possess immunoregulatory effects, via vascular remodelling, we speculate eribulin may induce reversal of EMT and this effect may contribute to prolonging OS. Moreover, we speculate that tumours with high-ALC levels in peripheral blood may reflect a favourable immune microenvironment that potentiates benefit from eribulin treatment.

We identified that high ALC (≥ 1500/µl) was a significant and independent predictor for longer OS in patients treated with eribulin through our evaluation of data from EMBRACE. However, we cannot exclude the possibility that treatment with prior chemotherapy may have influenced baseline ALC, despite exclusion of patients who had received previous treatment within 3 weeks of EMBRACE. Therefore, baseline ALC and NLR should be further evaluated in patients receiving first-line treatment with eribulin. Additionally, as this was a post hoc analysis, these results should be considered hypothesis generating. Considering this limitation, prospective studies or additional investigations are required to further validate the results of this report.

## Conclusions

In this hypothesis-generating analysis of EMBRACE, patients with high baseline ALC (≥ 1500/µl) showed longer OS with eribulin treatment, but not with TPC. Generally, ALC demonstrated potential as an immunological predictive index, hence, we suggest that longer OS in eribulin-treated patients is associated with modulation of the tumour microenvironment, such as the immune-regulation system. These results may be useful in selecting patients who may have greater OS benefits with eribulin. As ALC is a simple biomarker that can be evaluated without the need for additional invasive procedures, clinical utility of ALC may be important not only for predicting eribulin efficacy but also in considering biomarkers for use in combination therapy with ICIs.

## Electronic supplementary material

Below is the link to the electronic supplementary material.

Supplementary file1 (DOCX 1078 kb)

## References

[1] Halaven (eribulin mesylate) [prescribing information]. Woodcliff Lake, NJ: Eisai Inc.; 2017.

[2] Halaven 0.44 mg/ml solution for injection [summary of product characteristics]. Hertfordshire, UK: Eisai Europe Limited; 2019.

[3] Halaven [Japanese prescribing information]. Tokyo, Japan: Eisai Co., Ltd; 2016.

[4] Cortes J, O'Shaughnessy J, Loesch D, Blum JL, Vahdat LT, Petrakova K (2011). Eribulin monotherapy versus treatment of physician's choice in patients with metastatic breast cancer (EMBRACE): a phase 3 open-label randomised study. Lancet.

[5] Yoshida T, Ozawa Y, Kimura T, Sato Y, Kuznetsov G, Xu S (2014). Eribulin mesilate suppresses experimental metastasis of breast cancer cells by reversing phenotype from epithelial-mesenchymal transition (EMT) to mesenchymal-epithelial transition (MET) states. Br J Cancer.

[6] Funahashi Y, Okamoto K, Adachi Y, Semba T, Uesugi M, Ozawa Y (2014). Eribulin mesylate reduces tumor microenvironment abnormality by vascular remodeling in preclinical human breast cancer models. Cancer Sci.

[7] Kashiwagi S, Asano Y, Goto W, Takada K, Takahashi K, Noda S (2017). Use of tumor-infiltrating lymphocytes (TILs) to predict the treatment response to eribulin chemotherapy in breast cancer. PLoS ONE.

[8] Miyagawa Y, Araki K, Bun A, Ozawa H, Fujimoto Y, Higuchi T (2018). Significant association between low baseline neutrophil-to-lymphocyte ratio and improved progression-free survival of patients with locally advanced or metastatic breast cancer treated with eribulin but not with nab-paclitaxel. Clin Breast Cancer.

[9] Noh H, Eomm M, Han A (2013). Usefulness of pretreatment neutrophil to lymphocyte ratio in predicting disease-specific survival in breast cancer patients. J Breast Cancer.

[10] Ethier JL, Desautels D, Templeton A, Shah PS, Amir E (2017). Prognostic role of neutrophil-to-lymphocyte ratio in breast cancer: a systematic review and meta-analysis. Breast Cancer Res.

[11] Liu H, Zhang T, Ye J, Li H, Huang J, Li X (2012). Tumor-infiltrating lymphocytes predict response to chemotherapy in patients with advance non-small cell lung cancer. Cancer Immunol Immunother.

[12] Kocián P, Šedivcová M, Drgáč J, Cerná K, Hoch J, Kodet R (2011). Tumor-infiltrating lymphocytes and dendritic cells in human colorectal cancer: their relationship to KRAS mutational status and disease recurrence. Hum Immunol.

[13] Lee WS, Kang M, Baek JH, Lee JI, Ha SY (2013). Clinical impact of tumor-infiltrating lymphocytes for survival in curatively resected stage IV colon cancer with isolated liver or lung metastasis. Ann Surg Oncol.

[14] Araki K, Ito Y, Fukada I, Kobayashi K, Miyagawa Y, Imamura M (2018). Predictive impact of absolute lymphocyte counts for progression-free survival in human epidermal growth factor receptor 2-positive advanced breast cancer treated with pertuzumab and trastuzumab plus eribulin or nab-paclitaxel. BMC Cancer.

[15] Ueda S, Saeki T, Takeuchi H, Shigekawa T, Yamane T, Kuji I (2016). In vivo imaging of eribulin-induced reoxygenation in advanced breast cancer patients: a comparison to bevacizumab. Br J Cancer.

[16] Ito K, Hamamichi S, Abe T, Akagi T, Shirota H, Kawano S (2017). Antitumor effects of eribulin depend on modulation of the tumor microenvironment by vascular remodeling in mouse models. Cancer Sci.

[17] Zhao S, Yu W, Ukon N, Tan C, Nishijima KI, Shimizu Y (2019). Elimination of tumor hypoxia by eribulin demonstrated by ^18^F-FMISO hypoxia imaging in human tumor xenograft models. EJNMMI Res.

[18] Goto W, Kashiwagi S, Asano Y, Takada K, Morisaki T, Fujita H (2018). Eribulin promotes antitumor immune responses in patients with locally advanced or metastatic breast cancer. Anticancer Res.

[19] Li MO, Wan YY, Sanjabi S, Robertson AK, Flavell RA (2006). Transforming growth factor-beta regulation of immune responses. Annu Rev Immunol.

[20] Muto Y, Kitano S, Tsutsumida A, Namikawa K, Takahashi A, Nakamura Y (2019). Investigation of clinical factors associated with longer overall survival in advanced melanoma patients treated with sequential ipilimumab. J Dermatol.

[21] Ku GY, Yuan J, Page DB, Schroeder SE, Panageas KS, Carvajal RD (2010). Single-institution experience with ipilimumab in advanced melanoma patients in the compassionate use setting: lymphocyte count after 2 doses correlates with survival. Cancer.

[22] Noman MZ, Desantis G, Janji B, Hasmim M, Karray S, Dessen P (2014). PD-L1 is a novel direct target of HIF-1α, and its blockade under hypoxia enhanced MDSC-mediated T cell activation. J Exp Med.

[23] Taylor MA, Lee YH, Schiemann WP (2011). Role of TGF-β and the tumor microenvironment during mammary tumorigenesis. Gene Expr.

