# Supplementary material for: High absolute lymphocyte counts are associated with longer overall survival in patients with metastatic breast cancer treated with eribulin—but not with treatment of physician’s choice—in the EMBRACE study
Source: Breast Cancer. 2020 Mar 5;27(4):706–15. doi: 10.1007/s12282-020-01067-2 (PMC7297864; doi:10.1007/s12282-020-01067-2)
Supplement: Supplementary file 1 — Supplementary file1 (DOCX 1078 kb) [file 12282_2020_1067_MOESM1_ESM.docx]

**Electronic Supplementary Material**

**Supplemental Table 1.** Demographic and baseline characteristics by NLR group.

| **Characteristic, n (%)** | **Eribulin (*n*=475)** | | **TPC^a^ (*n*=238)** | |
| --- | --- | --- | --- | --- |
|  | **NLR <3**  **(*n*=230)** | **NLR ≥3**  **(*n*=245)** | **NLR <3**  **(*n*=114)** | **NLR ≥3**  **(*n*=124)** |
| Geographical region |  |  |  |  |
| North America/Western Europe/Australia | 130 (57) | 184 (75) | 68 (60) | 92 (74) |
| Eastern Europe | 70 (30) | 37 (15) | 31 (27) | 20 (16) |
| Latin America/South Africa | 30 (13) | 24 (10) | 15 (13) | 12 (10) |
| HER2 status |  |  |  |  |
| Positive | 40 (17) | 38 (16) | 16 (14) | 20 (16) |
| Negative | 163 (71) | 190 (78) | 89 (78) | 96 (77) |
| Unknown | 27 (12) | 17 (7) | 9 (8) | 8 (6) |
| Prior capecitabine treatment |  |  |  |  |
| Yes | 164 (71) | 188 (77) | 82 (72) | 99 (80) |
| No | 66 (29) | 57 (23) | 32 (28) | 25 (20) |
| ECOG performance status |  |  |  |  |
| 0 | 112 (49) | 92 (38) | 54 (47) | 46 (37) |
| ≥1 | 117 (51) | 146 (60) | 60 (53) | 75 (60) |
| Age group |  |  |  |  |
| <65 years | 197 (86) | 188 (77) | 95 (83) | 91 (73) |
| ≥65 years | 33 (14) | 57 (23) | 19 (17) | 33 (27) |
| ER status |  |  |  |  |
| Positive | 152 (66) | 169 (69) | 78 (68) | 86 (69) |
| Negative | 63 (27) | 71 (29) | 31 (27) | 36 (29) |
| Unknown | 15 (7) | 5 (2) | 5 (4) | 2 (2) |
| PgR status |  |  |  |  |
| Positive | 114 (50) | 128 (52) | 57 (50) | 59 (48) |
| Negative | 84 (37) | 101 (41) | 43 (38) | 54 (44) |
| Unknown | 32 (14) | 16 (7) | 14 (12) | 11 (9) |
| HR status |  |  |  |  |
| Positive | 157 (68) | 175 (71) | 84 (74) | 87 (70) |
| Negative | 54 (23) | 63 (26) | 25 (22) | 34 (27) |
| Unknown | 19 (8) | 7 (3) | 5 (4) | 3 (2) |
| Triple negative |  |  |  |  |
| Triple negative | 41 (18) | 47 (19) | 21 (18) | 28 (23) |
| Non-triple negative | 189 (82) | 198 (81) | 93 (82) | 96 (77) |
| Site of disease |  |  |  |  |
| Visceral disease | 188 (82) | 202 (82) | 88 (77) | 111 (90) |
| Non-visceral disease | 42 (18) | 42 (17) | 24 (21) | 13 (10) |
| Number of organs involved |  |  |  |  |
| ≤2 | 185 (80) | 166 (68) | 82 (72) | 73 (59) |
| >2 | 45 (20) | 78 (32) | 30 (26) | 51 (41) |
| Number of prior chemotherapy regimens |  |  |  |  |
| ≤3 | 121 (53) | 99 (40) | 56 (49) | 47 (38) |
| >3 | 107 (47) | 146 (60) | 57 (50) | 77 (62) |
| Number of prior chemotherapy regimens for locally advanced or metastatic disease |  |  |  |  |
| ≤3 | 185 (80) | 180 (73) | 83 (73) | 84 (68) |
| >3 | 45 (20) | 65 (27) | 30 (26) | 40 (32) |
| Refractory to taxanes^b^ |  |  |  |  |
| Yes | 173 (75) | 207 (84) | 91 (80) | 102 (82) |
| No | 57 (25) | 38 (16) | 23 (20) | 22 (18) |

^a^TPC was defined as any single-agent chemotherapy, or hormonal or biological therapy, approved for the treatment of cancer.

^b^Disease progression on or within 6 months of taxane treatment.

ECOG, Eastern Cooperative Oncology Group; ER, oestrogen receptor; HER2, human epidermal growth factor receptor-2; HR, hormone receptor; NLR, neutrophil-to-lymphocyte ratio; PgR, progesterone receptor; TPC, treatment of physician’s choice.

**Supplemental Table 2****.** Cut-off value of ALC as a predictive factor of the effect of eribulin on OS.

| **ALC Cut-off (/μl)** | **Treatment** | ***n*** | **Median (months)** | **HR (Eribulin/TPC)** | **95% CI (Lower)** | **95% CI (Upper)** | **Interaction *P*-value** |
| --- | --- | --- | --- | --- | --- | --- | --- |
| ≥1300 | Eribulin | 258 | 15.0 | 0.743 | 0.578 | 0.957 | 0.3982 |
|  | TPC | 131 | 11.4 |  |  |  |  |
| <1300 | Eribulin | 242 | 11.1 | 0.865 | 0.673 | 1.112 |  |
|  | TPC | 120 | 9.1 |  |  |  |  |
| ≥1400 | Eribulin | 234 | 15.7 | 0.641 | 0.490 | 0.840 | 0.0136 |
|  | TPC | 112 | 11.4 |  |  |  |  |
| <1400 | Eribulin | 266 | 11.1 | 0.993 | 0.783 | 1.258 |  |
|  | TPC | 139 | 10.1 |  |  |  |  |
| ≥1500 | Eribulin | 199 | 15.6 | 0.586 | 0.437 | 0.784 | 0.0031 |
|  | TPC | 92 | 11.4 |  |  |  |  |
| <1500 | Eribulin | 301 | 11.6 | 1.002 | 0.800 | 1.253 |  |
|  | TPC | 159 | 10.3 |  |  |  |  |
| ≥1600 | Eribulin | 172 | 15.7 | 0.578 | 0.418 | 0.799 | 0.0042 |
|  | TPC | 72 | 10.5 |  |  |  |  |
| <1600 | Eribulin | 328 | 11.8 | 0.964 | 0.780 | 1.193 |  |
|  | TPC | 179 | 10.7 |  |  |  |  |
| ≥1700 | Eribulin | 149 | 15.7 | 0.633 | 0.444 | 0.903 | 0.0326 |
|  | TPC | 62 | 10.5 |  |  |  |  |
| <1700 | Eribulin | 351 | 12.3 | 0.907 | 0.739 | 1.114 |  |
|  | TPC | 189 | 10.7 |  |  |  |  |
| ≥1800 | Eribulin | 130 | 14.9 | 0.629 | 0.425 | 0.931 | 0.0681 |
|  | TPC | 51 | 10.5 |  |  |  |  |
| <1800 | Eribulin | 370 | 12.5 | 0.873 | 0.715 | 1.066 |  |
|  | TPC | 200 | 10.7 |  |  |  |  |

ALC, absolute lymphocyte count; CI, confidence interval; HR, hazard ratio; OS, overall survival; TPC, treatment of physician’s choice.

**Electronic Supplementary Material**


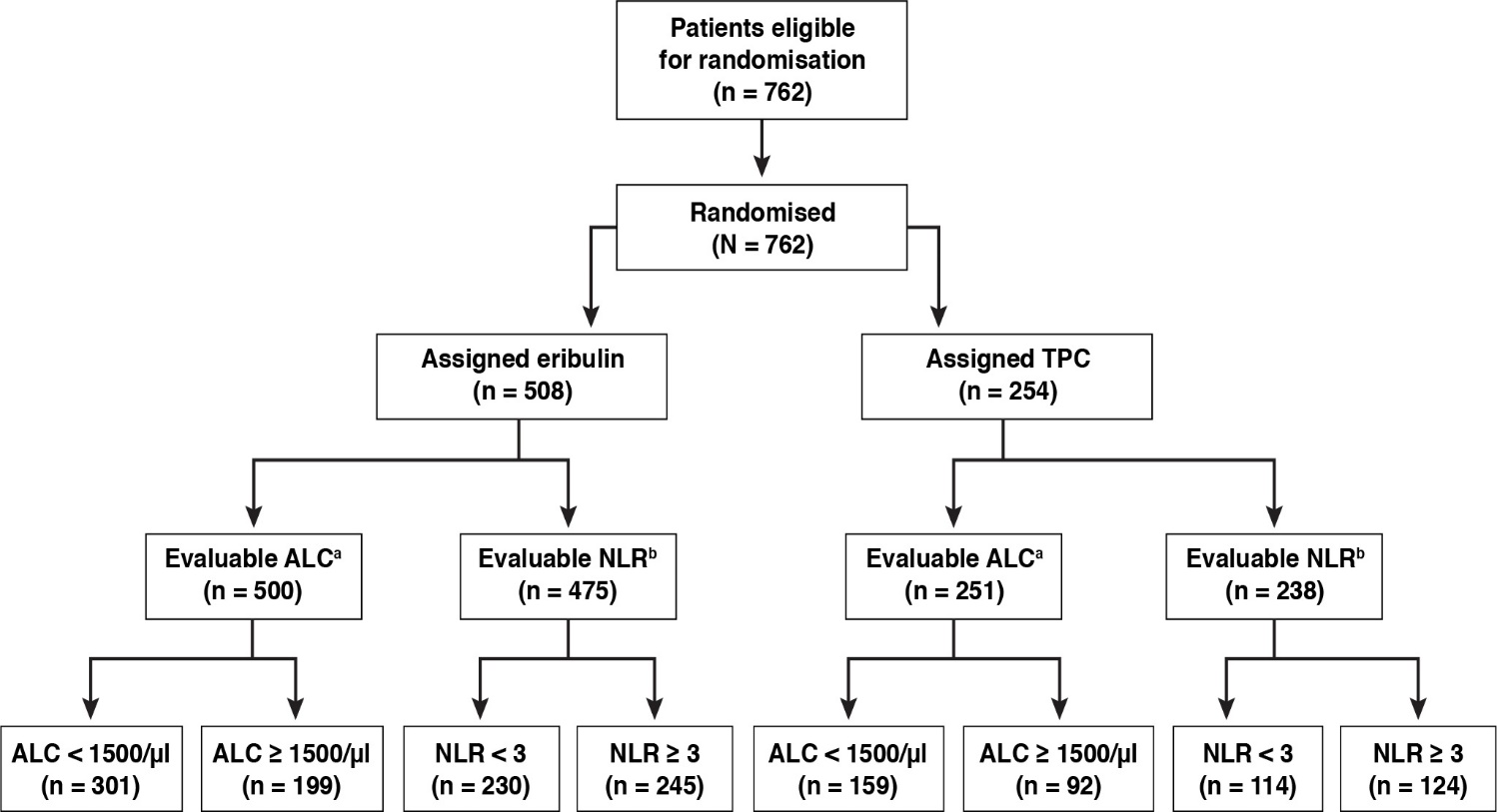
**Supplemental Fig. 1** CONSORT diagram of study population in EMBRACE

^a^Data from 8 patients in the eribulin arm and 3 patients in the TPC arm were omitted due to missing baseline ALCs.

^b^Data from 33 patients in eribulin arm and 16 patients in the TPC arm were omitted due to missing baseline NLRs.

ALC, absolute lymphocyte count; NLR, neutrophil-to-lymphocyte ratio; TPC, treatment of physician’s choice.

**Supplemental Fig. 2** Interaction between treatment and baseline ALC (<1500/µl/ ≥1500/µl) on PFS (Independent review)


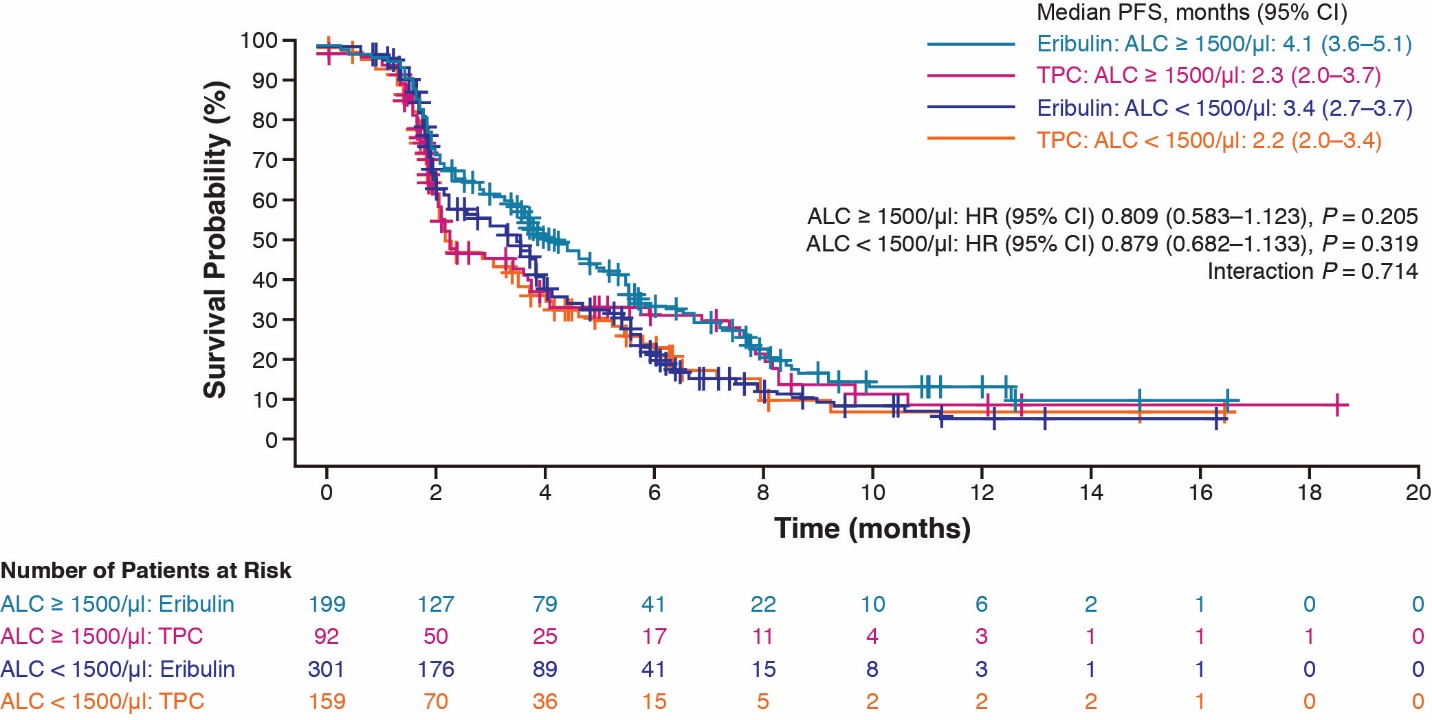


ALC, absolute lymphocyte count; CI, confidence interval; HR, hazard ratio; PFS, progression-free survival; TPC, treatment of physician’s choice.

**Supplemental Fig. 3** Interaction between treatment and baseline NLR (<3/≥3) on PFS (Independent review)


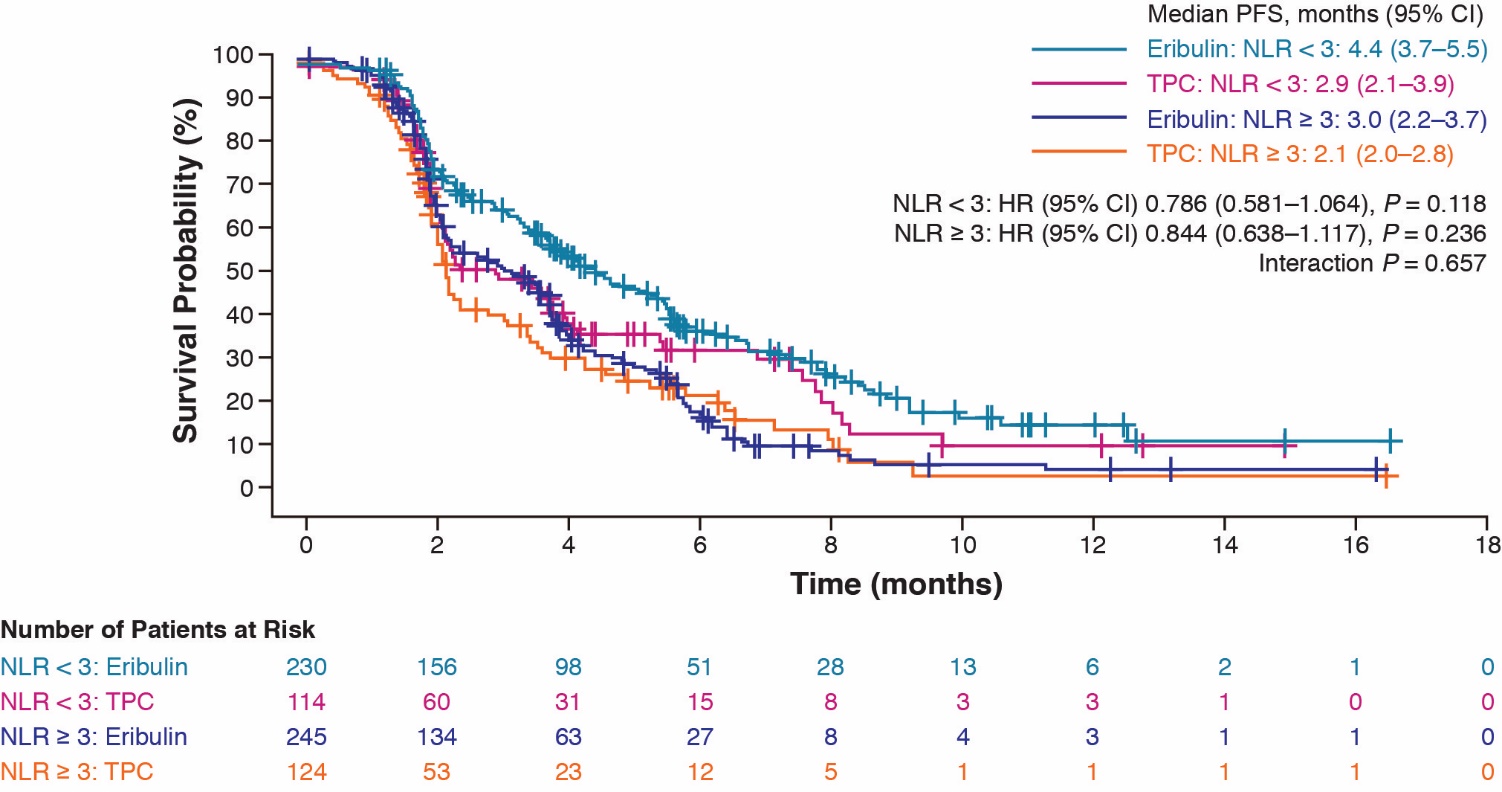


CI, confidence interval; HR, hazard ratio; NLR, neutrophil-to-lymphocyte ratio; PFS, progression-free survival; TPC, treatment of physician’s choice.

**Supplemental Fig. 4** Forest plot for baseline ALC (<1500/µl vs ≥1500/µl) effects on OS for the TPC arm


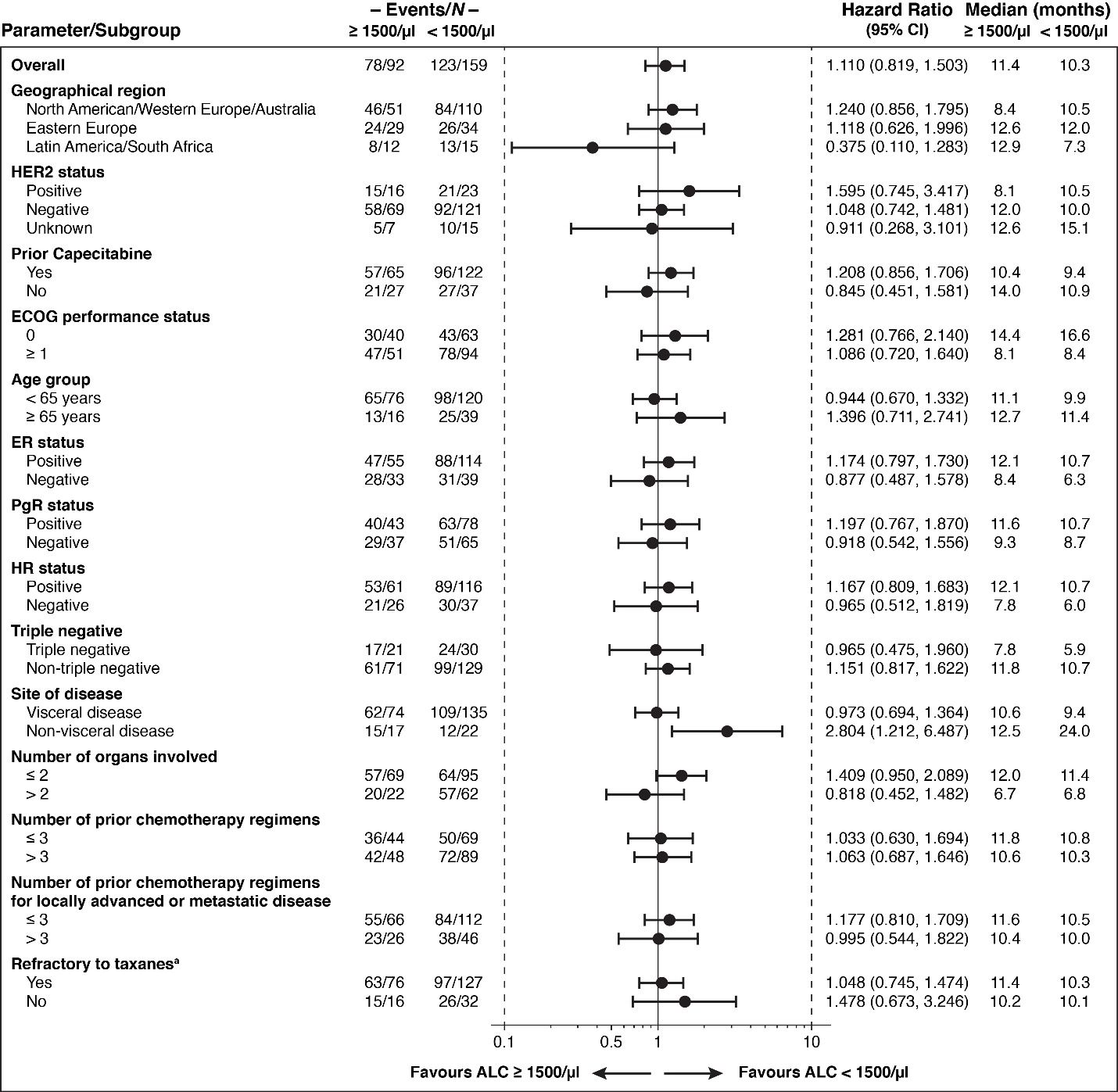


^a^Disease progression on or within 6 months of taxane treatment.

ALC, absolute lymphocyte count; CI, confidence interval; ER, oestrogen receptor, HR, hormone receptor; OS, overall survival; PgR, progesterone receptor; TPC, treatment of physician’s choice.
